# Assessing the nexus between knowledge management and firm performance: A data article

**DOI:** 10.1016/j.dib.2020.106283

**Published:** 2020-09-04

**Authors:** Mohammad J Adaileh, Muneer Alrawashdeh, Hamzah Elrehail, Khaled J Aladayleh

**Affiliations:** a Aqaba University of Technology, Aqaba, Jordan; b Middle East University, Amman, Jordan; c Skyline University College, Sharjah, United Arab Emirates; d Department of Engineering Projects, Universitat Politècnica de València, Valencia 46022, Spain

**Keywords:** Knowledge management, Firm performance, Medium-sized and large companies, Multi-group analysis, Group invariance, Jordan

## Abstract

This data article highlights the contingent role of company size in the relationship between knowledge management practices and firm performance. It also investigates the possible direct impact of these practices on performance. Data was collected from managers in large and medium-sized industrial companies in Jordan, using a self-administered questionnaire. 170 responses were obtained, 91 from medium-sized companies and 79 from large companies. Confirmatory factor analysis was used to ensure the validity and reliability of the measurement model. Multiple group structural equation modeling was used to check for significant differences in the path coefficients of the research model. The fitness indicators of the multi-group model showed that there was no significant difference in the interpretation of the measurement model; also, the path model was equivalent for both large and medium-sized companies. Testing the hypotheses showed that the application of knowledge has the greatest explanatory power for performance, whether in medium or large companies; however, for knowledge capturing the explanatory power on performance was only for medium-sized companies, and there was no effect on large companies. Moreover, the acquisition and sharing of knowledge had no statistically significant effect on performance in either group.

## Specifications Table

**Table:** 

Subject area	Management, business, knowledge management
More specific subject area	Knowledge management practices, firm performance
Type of data	Tables, figures, dataset, questionnaire
How data was acquired	By distributing questionnaires to managers in industrial companies in Jordan
Data format	Raw, analyzed, descriptive and statistical data
Parameters for data collection	To collect data, a self-administered questionnaire survey was conducted with Jordanian industrial companies known to have wide experience in the field of knowledge management and to have systems for managing their knowledge process. The questionnaire was designed to collect information about respondents’ demographic characteristics: company size, age of the company, manager's experience, qualifications, and job title.
Description of data collection	- Sample of managers in large and medium-sized industrial companies. - Company directors are considered to have greater insight in providing information related to knowledge management and performance practices. - The moderating effect of company size in knowledge management and performance is not well explored in the literature.
Data source location	Industrial companies located the capital of Jordan, Amman.
Data accessibility	http://dx.doi.org/10.17632/kjp4swr5f2.2

## Value of the Data

• This article is useful because it highlights the impact of knowledge management on firm performance; the contingent effect of company size is tested using multi-group structural equation modeling.
• This dataset will be valuable to companies, as it provides an appropriate background for all important aspects of knowledge management practices that increase performance.
• Few studies have investigated the contingent role of company size. The scale which has been developed and validated in this dataset can be used for further analysis, for example to measure the impact of knowledge management practices on organizational performance in different settings.
• This data article can be used by researchers, academics, and graduate students seeking to publish their research. It is also possible to use the methods of analysis used in this data article.

## 1 Data Description

In order to analyze data, IBM SPSS AMOS, V24 was used. Demographic information was generated by analyzing the frequencies of the variables company size, age of the company, the manager's experience, qualifications, and job title. See Table 1. Data screening was conducted by following the recommendations of [1,2]; multiple imputation was applied to questionnaires with missing values below 20%. Also, the observations farthest from the centroid (outliers) were determined based on Mahalanobis distance. Cases with missing values more than 20% were excluded in order to ensure a normal distribution of data.

**Table 1:** Demographic information of sample.

Characteristic		Frequency	(%)
Company size	Medium	91	53.5
	Large	79	46.5
Age of the company	Less than 5 years	3	1.8
	5 – less than 10 years	13	7.6
	10 – less than 15 years	121	71.2
	15 years and above	33	19.4
Manager's experience	Less than 5 years	17	10
	5 – less than 10	26	15.3
	10 – less than 15	89	52.4
	15 or more	38	22.4
Manager's qualification	BA	134	78.8
	Master Degree	22	12.9
	PhD	14	8.2
Manager's job title	IT Manager/Knowledge/Information/Design	86	50.6
	Human Resources Manager	14	8.2
	Operations/Manufacturing/Logistics	29	17.1
	Customer Relations/Public Relations	41	24.1

A valid and widely accepted measurement instrument was adopted from the above literature to design the questionnaire for data collection; the answers were recorded on a 5-point Likert scale [from strongly disagree (1) to strongly agree (5) to assess the managers’ attitude to knowledge management practices and firm performance. The questionnaire consisted of 27 items in five sections to measure capturing, sharing, acquiring and applying knowledge, and firm performance; see Table 2. Company size and other demographic variables were measured using a nominal scale. Confirmatory factor analysis was conducted on the dataset. Table 2 presents the results of statistical analysis of the construct items and the procedures for evaluating convergent validity and reliability. Table 3 is a correlation matrix of the variables to evaluate the discriminant validity. The main focus of this dataset is to test the causal impact of knowledge management practices on firm performance, exploring how the knowledge management elements used by companies of different sizes affect their performance. Company size is a moderator on the causality between knowledge management factors and firm performance. Table 4 lists the hypotheses tested in the total model of 170 companies. In Table 5, the results of multi-group analysis and testing of the hypotheses for both large and medium-sized companies are presented.

**Table 2:** Statistics of construct items.

Constructs	Measurement items	S.E.	Regression weights (C.R.)	Standardized regression weights	SMC	AVE/ SQRT (AVE)	CR	**α**
Knowledge capturing	KC1		1.000 (Fix)	0.701	0.491	0.479 (0.692)	0.73	0.72
	KC2	0.07	0.871(12.166*)	0.697	0.486			
	KC3	0.07	0.882(11.899*)	0.678	0.460			
Knowledge sharing	KS5		1.000 (Fix)	0.793	0.629	0.657 (0.810)	0.88	0.85
	KS1	0.06	1.133(19.939*)	0.864	0.746			
	KS2	0.06	1.231(20.48*)	0.883	0.780			
	KS4	0.07	0.968(14.978*)	0.687	0.472			
Firm performance	FP3		1.000 (Fix)	0.654	0.428	0.523 (0.723)	0.85	0.86
	FP4	0.07	1.027(14.712*)	0.68	0.462			
	FP5	0.08	1.045(13.215*)	0.735	0.540			
	FP6	0.09	1.234(13.706*)	0.772	0.596			
	FP7	0.09	1.16513.627*)	0.767	0.588			
Knowledge acquisition	KA1		1.000 (Fix)	0.758	0.575	0.57 (0.754)	0.84	0.88
	KA2	0.06	1.069(19.355*)	0.772	0.596			
	KA4	0.06	0.941(16.116*)	0.753	0.567			
	KA5	0.07	1.067(15.736*)	0.736	0.542			
Knowledge application	KAP4		1.000 (Fix)	0.682	0.465	0.611 (0.781)	0.86	0.85
	KAP3	0.08	1.174(14.77*)	0.787	0.619			
	KAP2	0.08	1.246(15.712*)	0.846	0.716			
	KAP1	0.08	1.179(15.047*)	0.803	0.645			

S.E.: Standard Error, C.R.: Critical Ratio, SMC: Squared Multiple Correlation.

AVE: Average Variance Extracted; SQRT (AVE): Square Root of Average Variance Extracted.

CR: Composite Reliability.

α: Cronbach's Alpha.

Values in parentheses are critical ratios and all the values are significant at (*p* < 0.01) level.

**Table 3:** Correlation matrix of variables.

Construct	Knowledge capturing	Knowledge sharing	Firm performance	Knowledge acquisition	Knowledge application
Knowledge capturing	1				
Knowledge sharing	0.473** (0.035)	1			
Firm performance	0.668** (0.039)	0.391** (0.037)	1		
Knowledge acquisition	0.461** (0.03)	0.553** (0.029)	0.341** (0.034)	1	
Knowledge application	0.455** (0.027)	0.401** (0.022)	0.537** (0.039)	0.646** (0.037)	1

Off-diagonal: Squared correlations between the constructs.

**Significant at 0.01.

In parentheses are numbers of standard error of the covariance.

**Table 4:** Research hypotheses testing (Total *N* = 170).

Hypothesis/Path	Standardized estimate	C.R.	*p*-value	Decision
Knowledge capturing –> Firm performance	0.256	2.627	0.009	Supported
Knowledge sharing –> Firm performance	−0.136	−1.254	0.21	Not supported
Knowledge acquisition –> Firm performance	0.118	1.025	0.305	Not supported
Knowledge application –> Firm performance	0.487	4.843	***	Supported

Note: ***p < 0.01.

**Table 5:** Multi-group analysis and testing hypotheses.

	χ^2^	df	p-value	GFI	AGFI	NFI	RMSEA	RMR
Unconstrained model (Group variant)/Saturated model								
Constrained model (Group invariant)								
Structural weights	1.903(<3.00)	4	0.754	0.996(>0.90)	0.996(>0.90)	0.997(>0.90)	0.000(0.865)	0.017(<0.03)
Structural covariance	11.209	14	0.67	0.976(>0.90)	0.948 (>0.90)	0.984(>0.90)	0.000(0.905)	0.025(<0.03)
Structural residuals	11.583	15	0.71	0.975(>0.90)	0.95 (0.90)	0.983(0.90)	0.000(0.927)	0.027(<0.03)


Hypothesis (Path)	Medium (*N* = 91) Standardized estimate	C.R.	*p*-value	Decision	Large (*N* = 79) Standardized estimate	C.R.	*p*-value	Decision
Knowledge capturing –> Firm performance	0.303	2.394	0.017*	Supported	0.212	1.392	0.164	Not supported
Knowledge sharing –> Firm performance	−0.089	−0.622	0.534	Not Supported	−0.242	−1.433	0.152	Not supported
Knowledge acquisition –> Firm performance	0.075	0.515	0.607	Not Supported	0.221	1.147	0.251	Not supported
Knowledge application –> Firm performance	0.479	3.711	**	Supported	0.486	2.977	0.003**	Supported

Notes: The numbers in parentheses are *p*-value for RMSEA, all *p*-value for RMSEA are insignificant, also, the numbers in other parentheses mean cut-off threshold for accepting the model.

Notes: *p < 0.05, **p < 0.01.

## 2 Experimental Design, Materials and Methods

The measurement model's reliability and validity were verified. The confirmatory factor analysis was used to confirm the conceptual model which was built on theory. As a result, some items with low factor loading, less than 0.60 in each construct, and with Squared Multiple Correlation (SMC) less than 0.4, were eliminated. Two items were removed from knowledge capturing and firm performance, and one from knowledge sharing, knowledge acquisition, and knowledge application, resulting in 20 items (see Table 2). Removing weak factor loading resulted in good model fitness indices: ***χ*^2^**/df = 2.893, RMR = 0.035, GFI = 0.879, AGFI = 0.845, CFI = 0.930, NFI = 0.898, IFI = 0.931, TLI = 0.917, RMSEA = 0.066; this indicated acceptable fitness of the measurement model [3,4].

Reliability, convergent validity and discriminant validity of the measurement model were checked. Cronbach's α, composite reliability and average variance extracted (AVE) confirmed reliability and convergent validity. The factor loading for all items were found to be significant at the *p* < 0.01 level; Cronbach's α ranged from 0.72 to 0.88 for all constructs, indicating internal consistency. Composite reliability for all latent constructs ranged from 0.73 to 0.88. The AVE did not fall below 0.50 except for a few number of items that made up the capturing knowledge construct. Fornell and Larcker [5] suggest that that this is not a problem when the composite reliability values are above 0.70, so it can be concluded that the measurement model had adequate convergent validity (see Table 2). Table 3 discriminant validity is presented [5,6]; when the values of the square root of AVE are higher than all of the squared values within the correlation matrix, it can be concluded that discriminant validity has been achieved. Comparing the square root of AVE in Table 2 with the values of the squared correlation coefficients in Table 3, discriminant validity of all constructs in the measurement model is confirmed.

Structural equation modeling was performed to test the hypotheses, and the path model was specified to measure the impact of the four knowledge management practices on firm performance. The structural path model presented is a suitable fit as the model is saturated, therefore no fitness indices were reported. The results of testing the hypotheses for the total model of both groups (medium and large companies) in Table 4 indicate that knowledge capturing and application are both statistically significant, with standardized estimates (β) for knowledge capturing, β = 0.256, C.R. = 2.627, (*p* < 0.05), and for knowledge application, β = 0.487, C.R. = 4.843, (*p* < 0.01). Therefore, the proposed hypotheses that knowledge capturing and sharing affect firm performance were supported. On the other hand, hypotheses for the impact of knowledge sharing and acquisition on firm performance for all groups were not supported.

The main concern of this dataset is to investigate whether differences in company size affect knowledge management practices in industrial companies in Jordan. A multi-group SEM was conducted accordingly in order to check for significant differences in the path model according to company size, following the suggestion of [7], [8], [9], [10]. Table 5 shows the model fitness for the two separate groups of companies. As shown, all fitness indices met the acceptance level, and multi-group analysis was performed to check for equivalence in structural weights, structural covariance, and structural residuals. χ2 was found to be insignificant for these factors, revealing no difference between the groups’ measurement items as indicated by Jahmani et al. [11] and Taamneh et al. [12]. Other fitness indices appeared good, reaching the acceptable levels (see Table 5). Therefore, the items selected do not differ between the two groups. This means that this scale is appropriate to measure the impact of knowledge management practices on firm performance in both large and medium-sized companies, as stated by Byrne et al. [8].

The explanatory power of knowledge application in firm performance appeared to be higher for both groups; the path model for the effect of knowledge application on firm performance was significant β = 0.479, C.R. = 3.711, (*p* < 0.01) for medium companies, and also for large companies, β = 0.486, C.R. = 2.977, (*p* < 0.01). The impact of knowledge capturing on firm performance differed, however: the path coefficients for medium companies were β = 0.303, C.R. = 2.394, (*p* < 0.05) and large companies β = 0.212, C.R. = 1.392, (*p* > 0.05) but the difference was insignificant. As for the other paths (Knowledge Sharing –> Firm Performance, Knowledge Acquisition –> Firm Performance), there was no statistically significant difference between the medium and large company groups.

Finally, the impact of knowledge sharing on performance was not supported, and in both groups sharing knowledge may negatively affect the performance of the organization, especially in the case of sharing critical knowledge. This leads to a loss of competitive advantage, and necessarily influences the level of performance. This finding is supported by many researchers [13], [14], [15], [16], [17], [18]. The main objective of this data is to test the invariance of the measurement model for both large and medium-sized companies. As most knowledge management practices occur in such companies, this suggests that small companies practice limited knowledge management. Failure to support the hypotheses does not necessarily mean that there is a deficiency in defining the study problem. Moreover, the rejection indicates that there is a need for companies to support these practices. As for the methods of testing these hypotheses, the researchers assert that regression methods, whether using path analysis or stepwise regression, make no difference to the results. In general, medium or large companies need to support areas that do not affect performance, such as knowledge capture, knowledge sharing and knowledge acquisition.

## 3 Academic and managerial implications of this data article

This article has several implications for the practice and academic study of management. First, managers in large and medium companies must recognize the great benefits that knowledge management brings in order to achieve high levels of performance. Second, the development of effective systems for knowledge management practices necessarily involves achieving a competitive advantage which leads to improved levels of performance. Finally, organizations of different sizes must consider the application of knowledge, especially sensitive knowledge, as this has the greatest explanatory power on performance. For academics, this data article provides suitable guidance for researchers, PhD and Masters students in the field of management on how to analyze their data. The use of structural equations, especially for multiple groups, is highly recommended by many researchers.

## Ethics Statement

Informed consent was obtained from the respondents of the study and this research study was approved by the Middle East University internal review board.

## Declaration of Competing Interest

None.

## Appendix Supplementary materials

Image, application 1

Image, application 2
