# Supplementary material for: Assessing the nexus between knowledge management and firm performance: A data article
Source: Data Brief. 2020 Sep 4;32:106283. doi: 10.1016/j.dib.2020.106283 (PMC7494479; doi:10.1016/j.dib.2020.106283)
Supplement: Supplementary file 2 [file mmc2.docx]

| **Appendix. Measurement scale** | |
| --- | --- |
| Code | Measurement Items |
|  | **Knowledge Capturing** |
| KC1 | The company collects information about market conditions / consumers / products / supply and demand. |
| KC2 | The company collects information about customer preferences within the markets it serves. |
| KC3 | The company collects information about competitors in the market |
|  | **Knowledge Sharing** |
| KS5 | The company shares the acquired knowledge with all departments. |
| KS1 | The company’s senior management monitors the process of sharing the knowledge with other parties in the supply chain. |
| KS2 | The company provides knowledge related to markets, products, consumers, product design to all parties in the supply chain. |
| KS4 | The company shares knowledge about new products and services with all parties in the supply chain. |
|  | **Firm Performance** |
| FP3 | The products and services the company provides create a higher value for customers. |
| FP4 | The company offers competitive prices better than competitors. |
| FP5 | We have time to market products and services below the industry average. |
| FP6 | We are able to develop new products and services quickly. |
| FP7 | We are able to compete based on the quality of our products and services |
|  | **Knowledge Acquisition** |
| KA1 | The company has solid policies to preserve knowledge assets |
| KA2 | We adopts effective knowledge systems for obtaining information from inside and outside the company. |
| KA4 | The company has a knowledge-based system for absorbing all knowledge sources |
| KA5 | Knowledge engineering process include all technical, scientific and social aspects in the company |
|  | **Knowledge Application** |
| KAP4 | The company uses the knowledge gained in its operations |
| KAP3 | The company uses the accumulated knowledge in order to avoid making mistakes in the markets, providing products and services |
| KAP2 | The company uses its knowledge assets to make decisions about the problems it faces in the markets |
| KAP1 | The knowledge obtained from the markets contributes to the development of new products and services |
